# Management and outcomes of advanced hemangioendothelioma at a medical oncology clinic in an Indian tertiary care center

**DOI:** 10.2144/fsoa-2021-0132

**Published:** 2023-02-03

**Authors:** Ghazal Tansir, Sameer Rastogi, Adarsh Barwad, Rajni Yadav, Shamim Ahmed Shamim, Ekta Dhamija, Rambha Pandey, Rakesh Garg, Shakti Shrivastava

**Affiliations:** 1 Department of Medical Oncology, BRA IRCH, All India Institute of Medical Sciences, New Delhi, 110029, India; 2 Sarcoma Medical Oncology Clinic, All India Institute of Medical Sciences, New Delhi, 110029, India; 3 Department of Pathology, All India Institute of Medical Sciences, New Delhi, 110029, India; 4 Department of Nuclear Medicine, All India Institute of Medical Sciences, New Delhi, 110029, India; 5 Department of Radiodiagnosis, All India Institute of Medical Sciences, New Delhi, 110029, India; 6 Department of Radiation Oncology, All India Institute of Medical Sciences, New Delhi, 110029, India; 7 Department of Oncoanesthesia & Palliative Medicine, All India Institute of Medical Sciences, New Delhi, 110029, India

**Keywords:** hemangioendothelioma, rare tumors, sarcoma, tyrosine kinase inhibitors, vascular tumors

## Abstract

**Aim:**

Hemangioendotheliomas (HEs) are malignant vascular tumors with sparse descriptions in literature owing to their rarity.

**Study design:**

Ours is a retrospective study among patients of advanced HEs registered between September 2015 and April 2021.

**Results:**

There were 13 patients with median age 34.6 (range: 4–69 years), male preponderance (69%) and predominant subtype of epithelioid HE (76.9%). Common primary sites were viscera (46.2%) and bone (30.8%). Tyrosine kinase inhibitors (TKIs) yielded objective responses in 30% patients whereas chemotherapy only produced disease stabilization in 7.7%.

**Conclusion:**

We recognize an aggressive subset of HEs with manifestations such as acute liver failure and splenic rupture. Currently no biomarkers predict the efficacy of TKIs over chemotherapy; however, TKIs showed promising outcomes in this series.

## Plain language summary

Hemangioendotheliomas are rare malignant tumors with vascular origin. This retrospective study on 13 patients displays a male preponderance, the with epithelioid subtype being the commonest in the study population. The disease also showed aggressive manifestations such as acute liver failure and ruptured spleen. The viscera and bone were the commonest primary locations in 46.2 and 30.8% patients, respectively. Treatment with tyrosine kinase inhibitors showed a promising 30% response rate while chemotherapy showed stabilization of disease in 7.7% patients.

## Tweetable abstract

A retrospective study by @doctorneeghzl, @samdoc_mamc *et al.* on 13 patients of hemangioendothelioma, shows prominence of epithelioid subtype with primary in viscera. A 30% response with tyrosine kinase inhibitors shows promise #raretumors

Hemangioendothelioma (HE) is a low-grade malignant vascular neoplasm of borderline biologic behavior [1]. This tumor arises from the vascular endothelium, with activity that of between benign hemangioma and malignant angiosarcoma. It is an extremely rare entity comprising of only less than 1% of vascular tumors [2].

HE has a wide spectrum of presentations based on its different pathological subtypes. These subtypes are: epithelioid; papillary intralymphatic; retiform; Kaposiform; pseudomyogenic; and composite [3,4]. The most common subtype is epithelioid hemangioendothelioma (EHE) followed by the Kaposiform type. Immunohistochemistry (IHC) suggests a positivity of endothelial specific markers, namely, CD31, Fli-1, ERG, CD34 and factor VIII. Other non specific positive markers are cytokeratin and vimentin [5,6].

EHE affects patients of both sexes, with a slight female predominance [7]. Median age of onset of EHE is 36 years old, ranging widely from 7 to 83 years [8,9]. As per Hemangioendothelioma Epithelioid Hemangioendothelioma and Related Vascular Disorders (HEARD) Support Group [10], the most common primary site of EHE is liver alone in 21%, lung and liver combined in 18%, bone in 14% and lung in 12%.

The Kaposiform hemangioendothelioma (KHE) subtype has male predominance [11] and is common in infants with median age at diagnosis of 4 months [12]. It is associated with higher local aggressiveness and regional infiltration with no potential for metastasis. There is an association with a severe consumptive coagulopathy termed Kassabach–Merritt phenomenon in 70% patients [13]. Approximately 75% of the patients of KHE have cutaneous manifestations such as erythematous papules, plaques, nodules or indurated masses and the most common affected site is extremity [14].

Pseudomyogenic HE is a male predominant subtype with median age of 42 (range: 10–82 years) while retiform HE has a mean age of 36 years with female predominance [15,16]. Most common primary sites of both subtypes are extremities, head neck regions and trunk. Both these subtypes have indolent biological behavior with a higher rate of local recurrence with rare distant metastases [16,17].

HE is often misdiagnosed or diagnosed late, as 50–76% patients may be asymptomatic at disease onset. The prognosis of EHE is better than that of angiosarcoma with mean survival duration of 4.6 years, ranging between 6 months and 24 years [18].

Management options of HE depend upon the site and extent of disease. They include surgical management of the lesions amenable to resection, with negative surgical margins achieving the best outcomes in localized disease [19]. Liver transplant is also an option in hepatic epithelioid HEs with disease localised to the liver with the caveat of prolonged immunosuppression [20]. Advanced HEs are challenging to treat and systemic therapy has been employed for unresectable or metastatic disease with variable results. Apart from conventional chemotherapy, various tyrosine kinase inhibitors (TKI) are now being used and offer a newer option in management. Owing to the vascular origin of the disease and the vital role of angiogenesis in its etiopathogenesis, the VEGF pathway acts as a relevant target [21,22]. An indicator of the angiogenic dysregulation in HE is the VEGF expression found on IHC of hepatic EHE patients, further supporting the use of TKIs that target the VEGF-VEGF Receptor pathways. Indian data regarding HE is scarce, with no case series reported till date as is the case with majority of rare tumors [23].

## Materials & methods

This is a retrospective study evaluating the patients with localized or advanced HEs registered in sarcoma medical oncology clinics between September 2015 and April 2021 with follow-up until October 2021. Pathology of the cases were reported and/or reviewed by the expert histopathologists (AB and RY) at our institution. The management plans for the patients were devised as per discussion in multidisciplinary clinics. After clearance from the Institute Review Board, patient data were evaluated through hospital records including age, sex, primary site, metastatic sites, histopathology and IHC, therapy administered, response rate and outcomes. Statistical analysis was done through SPSS 26 (SPSS, IL, USA). Nominal data are provided as number (%) and continuous data as median and mean values as applicable. Progression free survival (PFS) was calculated from the date of initiation of treatment to the first date of documented progressive disease or death from any cause. OS was calculated from the date of diagnosis to death from any cause and patients alive or lost to follow-up will be censored.

## Results

**Figure 1.**
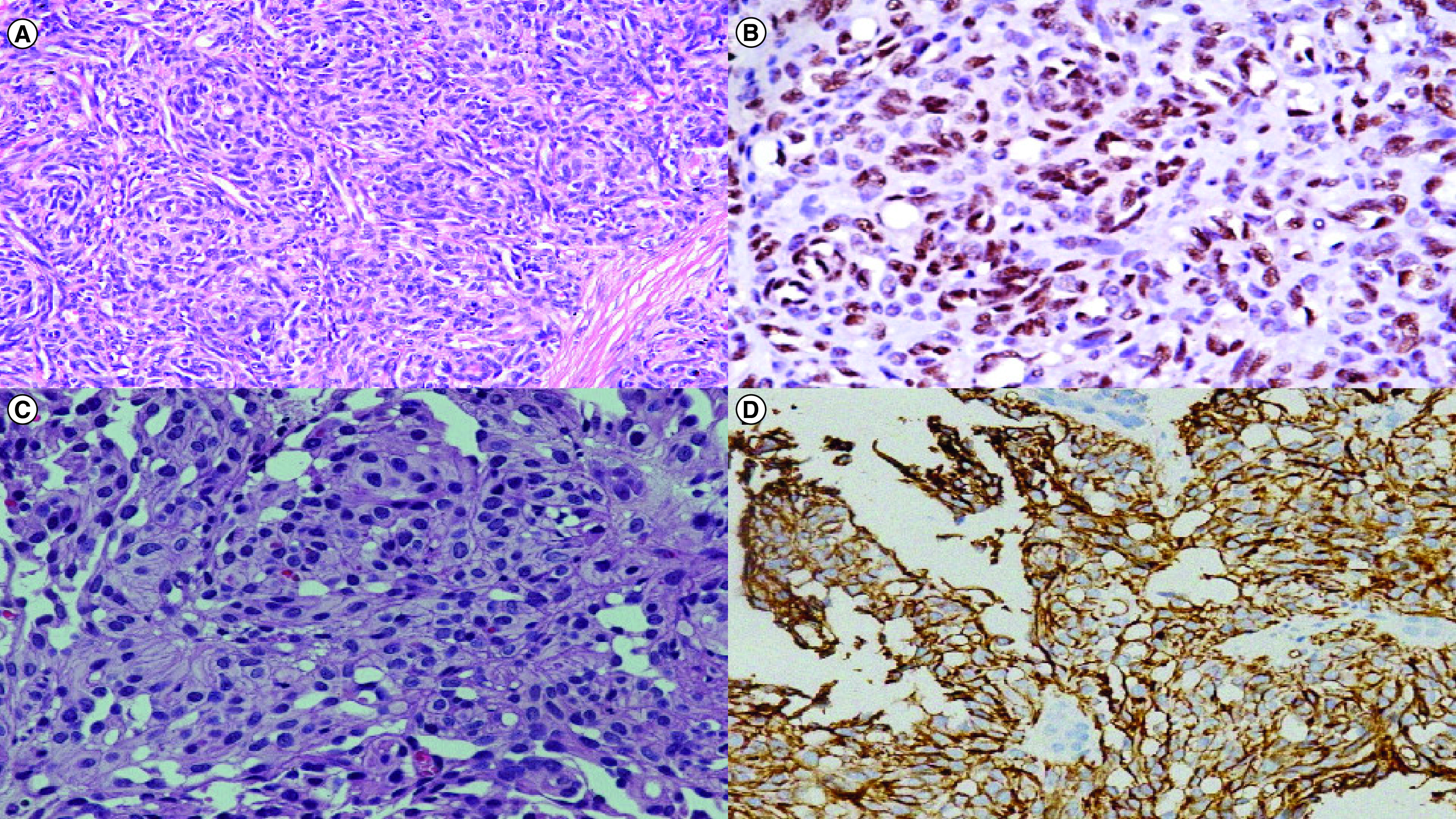
Histopathological findings of hemangioendothelioma. **(A)** H&E image in 100x magnification showing representative photomicrograph of the case of Kaposiform hemangioendothelioma with cannonball pattern of arrangement and slit like vascular spaces lined by mildly pleomorphic endothelial cells. There is no significant atypia and no mitotic figures. **(B)** Immunostain for FLI-1 showing nuclear positivity in the endothelial cells. **(C)** H&E image in 200x magnification showing representative photomicrograph from a case of epithelioid hemangioendothelioma with cells with epithelioid morphology showing mild to moderate pleomorphism with abundant eosinophilic cytoplasm. Many cells show presence of intracytoplasmic lumina characteristic of endothelial cells. **(D)** Immunostain for CD31 antigen showing membrano-cytoplasmic positivity in endothelial cells.

We analyzed a total of 13 patients with the most prevalent histopathological subtype being EHE (n = 10; 77%). The other subtypes, namely, Kaposiform (Figure 1), retiform and pseudomyogenic HE, were second-most common (n = 1; 7.6% each). Our patient population comprised of nine males (69%) and four females (31%). The median age at presentation overall was 34.6 (age range: 4–60 years). Further details of epidemiologic findings and clinical presentation of EHE and other subtypes are further described in Table 1.

**Table 1.** Clinico-epidemiological profile of cases under study.

	All subtypes of hemangioendothelioma	EHE only
Median age, years (range)	34.5 (4–60)	32.5 (8–69)
Primary (n, %)	Liver (4, 30.7%) Vertebra (4, 30.7%) Spleen (2, 15.3%) Tibia (1, 7.6%) Chest wall (1, 7.6%) Paravertebral (1, 7.6%)	Liver (4, 40%) Vertebrae (4, 40%) Paravertebral (1, 10%) Spleen (1, 10%)
Pattern of metastasis (%)	Lymph node (6, 46.1%) Bone (6, 46.1%) Lung (3, 23.2%) Liver (2, 15.3%) Others (30.7%)	Lymph node (6, 60%) Bone (5, 50%) Lung (3, 10%) Pancreas (1, 20%) Pleural fluid (1, 10%) Pleura (1, 10%) Liver (1, 10%) Orbit (1, 10%)
Sex distribution (n, %)	Male (9, 69%) Female (4, 31%)	Male (6, 60%) Female (4, 40%)
Symptoms (n, %)	Pain (10, 76.9%)^†^ Swelling (1, 7.6%) Paraparesis (1, 7.6%) Asymptomatic (1, 7.6%)	Pain (8, 80%) Paraparesis (1, 10%) Asymptomatic (1, 10%)
Other events		Acute liver failure (1, 10%)

† Splenic rupture with hemoperitoneum in one patient requiring splenectomy.

EHE: Epithelioid hemangioendothelioma.

The disease was metastatic in 77% patients (n = 10), locally advanced unresectable in 15.3% patients (n = 2) non metastatic in 7.7% patients (n = 1). The most common primary site was viscera (46.2%; n = 6) followed by bone (30.8%; n = 4) and soft tissue (23.1%; n = 3). The most common sites of metastases were lymph node (46.1%; n = 6), bone (46.1%; n = 6), lung (23.2%; n = 3), liver (15.3%; n = 2) and other rarer sites as mentioned in Table 1.

The EHE subtype comprised of 60% males (n = 6) and 40% females (n = 4), with a median age of 32.5 (age range: 8–69 years). The disease was metastatic in nine patients (n = 9) and localized/locally advanced in 10% (n = 1). The viscera (liver and spleen) were the most frequent primary site (n = 5; 50%) followed by bone (n = 4; 40%). The most common sites of metastases were lymph node in 60% (n = 6), bone in 50% (n = 5), lung in 30% (n = 3) and others. EHE occurred in the postpartum period in one patient, while it developed post completion of chemotherapy for mediastinal germ cell tumor in another patient. A detailed description of each case including presentation, histology, treatment given and outcomes has been elucidated in Table 2.

**Table 2.** Details of patients in the study including clinical presentation, histologic subtype, treatment given with response and outcomes.

S. no.	Age (years)/sex	Primary site	Histology	Clinical presentation/stage	Metastatic sites	First-line therapy	Best response/duration of therapy (months)	Second-line therapy	Best response/duration of therapy (months)	Current status
1	22/female	Liver	EHE	Postpartum abdominal pain/M	Lung, lymph node	Refused therapy				Deceased
2	45/male	Vertebra	EHE	Back pain/M	Lung, lymph node, bone	Paclitaxel + Bevacizumab	PD/4			Deceased
3	34/male	Liver	EHE	Abdominal pain/M	Bone, lymph node	Sorafenib 400 mg twice daily^†^	SD/36			Alive
4	27/male	Liver	EHE	Abdominal pain/M	Bone, lymph node, pancreas	Pazopanib 800 mg once daily^†^	SD/15			Alive
5	15/male	Spleen	EHE	Detected in mediastinal GCT survivor/M	Liver, lung	Pazopanib 800 mg once daily^†^	PD/4	Paclitaxel^†^	SD/27	Alive
6	60/male	Soft tissue (paravertebral)	EHE	Back pain/M	Pleural fluid, pleural deposit, lymph node	Gemcitabine + paclitaxel	PD/1	Pazopanib 800 mg once daily^†^	PR/27	Alive
7	8/male	Vertebrae	EHE	Paraparesis/M	Bone, orbit	Vinblastine^†^	PD/1	Sorafenib 100 mg once daily^†^	PR/23	Alive
8	69/female	Vertebrae	EHE	Back pain/M	Bone	Pazopanib 400 mg once daily^†^	PD/5			Deceased
9	31/female	Soft tissue (abdomen)	EHE	Abdominal pain/NM		Pazopanib 400 mg once daily^†^	TBA/1			Alive
10	49/female	Liver	EHE	Abdominal pain/M	Lymph node	Thalidomide 100 mg once daily^‡^	PD/3	Pazopanib 800 mg once daily^†^ reduced to 400 mg once daily	CR/62	Alive
11	4/male	Tibia	KHE	Leg swelling and pain/LA		Prednisone in slow tapering doses over 20 months^†^	CR/44 [only propranolol for 24 months]			Alive
12	35/male	Chest wall	PMHE	Chest pain/LA		Pazopanib 400 mg once daily^†^	PD/1 month	Doxorubicin	PD/2	Deceased
13	52/male	Spleen	RHE	Acute pain abdomen due to splenic rupture and hemoperitoneum/M	Liver, bone	Gemcitabine + paclitaxel^†^	PD/2 months	Pazopanib 600 mg once daily^†^	PD/less than 1	Deceased

† Propranolol given along with the drug regimen at median dose 80 mg daily.

‡ Celecoxib given along with the drug regimen at dose of 200 mg twice daily.

CR: Complete response; EHE: Epithelioid hemangioendothelioma; GCT: Germ cell tumor; KHE: Kaposiform hemangioendothelioma LA: Locally advanced unresectable; M: Metastatic; NM: Non metastatic; PD: Progressive disease; PR: Partial response; PMHE: Pseudomyogenic hemangioendothelioma; RHE: Retiform hemangioendothelioma; SD: Stable disease; TBA: To be assessed.

All surgical treatments for HE were performed in five patients (38.4%) at outside hospitals following which the diagnosis of HE was made. Debulking surgery was done for three patients (epithelioid, pseudomyogenic and Kaposiform subtypes) while the other two underwent orthotopic liver transplantation plus metastasectomy of costal metastasis and splenectomy respectively (epithelioid and retiform subtypes respectively).

TKIs were used in six out of 13 (46.1%) patients in first line and four out of six (66%) patients in second line. The two TKIs used among our patients were pazopanib (80%) and sorafenib (20%) in combination with propranolol (median dose 80 mg daily) in 100% cases.

Conventional chemotherapeutic agents were used in large volume or organ threatening disease and comprised of gemcitabine plus paclitaxel (n = 2), bevacizumab plus paclitaxel (n = 1), single-agent paclitaxel (n = 1), vinblastine (n = 1), doxorubicin (n = 1), as summarized in Table 3. The TKIs were started at a lower dose as per our prior published experience of higher toxicities in Indian patients at lower TKI dosages. The patients who continued to receive reduced doses were decided based on physicians' discretion and/or who experienced grade 3 or 4 toxicities with the full doses. Propranolol was added to chemotherapy in 60% and to TKIs in 100% patients, at a median dose of 80 mg daily. Table 2 elucidates the maximum doses of TKIs received by our patients. The clinical scenarios where chemotherapy was administered were rapidly progressive and painful soft tissue mass, vision-threatening orbital metastases, acute onset paraparesis and splenic rupture with impending hepatic rupture. The patient with postpartum EHE refused treatment and passed away due to disease progression. Further details of the various treatment regimens used across all lines of therapy are provided in Table 3.

**Table 3.** Details of therapy received, toxicity and survival outcomes.

	First line	Second Line
Medical treatment regimens across lines (n)	**TKI** Pazopanib + propranolol (5) Sorafenib + propranolol (1)	Pazopanib + propranolol (3) Sorafenib + propranolol (1)
	**Others** Prednisone + propanolol (1) Thalidomide + celecoxib (1)	
	**Chemotherapy** Gemcitabine + paclitaxel^†^ (2) Paclitaxel + Bevacizumab (1) Vinblastine + Propranolol (1)	Doxorubicin (1) Paclitaxel + propranolol (1)
Response at first assessment (%)	CR – 8.3% PR – 0% SD – 16.6% PD – 66.6%	CR – 16% PR – 33% SD – 16% PD – 33%
Toxicity (n, %)	Febrile neutropenia with vinblastine (1, 7.7%) Neuropathy with paclitaxel (1, 7.7%)	Grade III hand foot reaction (1; 10%)

† Combined with propranolol in one patient.

CR: Complete response; PD: Progressive disease; PR: Partial response; SD: Stable disease; TKI: Tyrosine kinase inhibitor.

**Figure 2.**
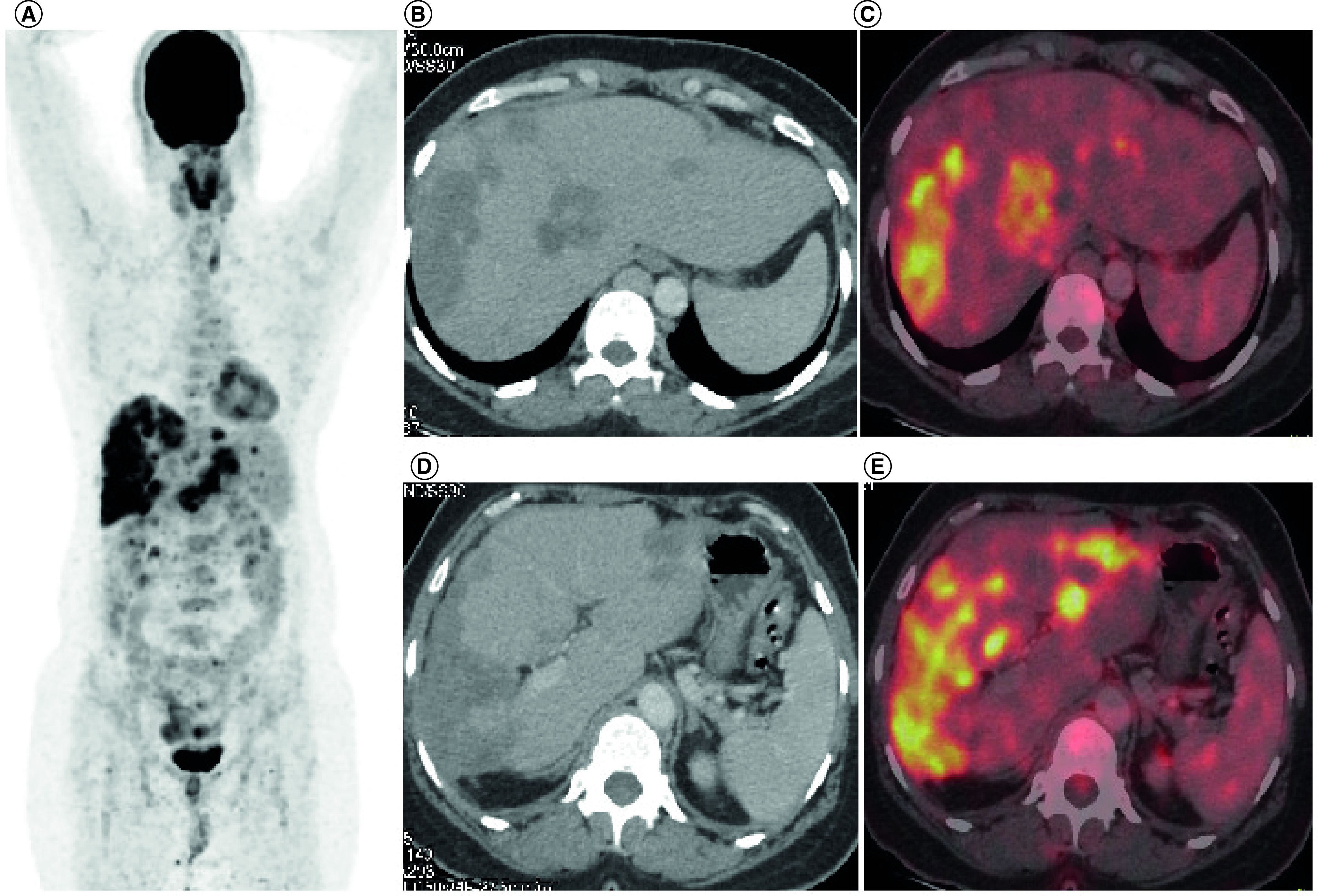
Fluorodeoxyglucose-positron emission tomography images of a 49-year-old female patient of metastatic hepatic epithelioid hemangioendothelioma. This scan represents the radiological findings prior to initiation of pazopanib-based therapy for this patient. **(A)** MIP image FDG PET-CT showing increased tracer uptake in the hepatic region. **(B & D)** Axial CT abdomen showing confluent hypodense lesions in both the lobes of liver. **(C & E)** There is increased tracer uptake noted at the liver lesions in the fused PET-CT images. MIP: Maximum intensity projection.

**Figure 3.**
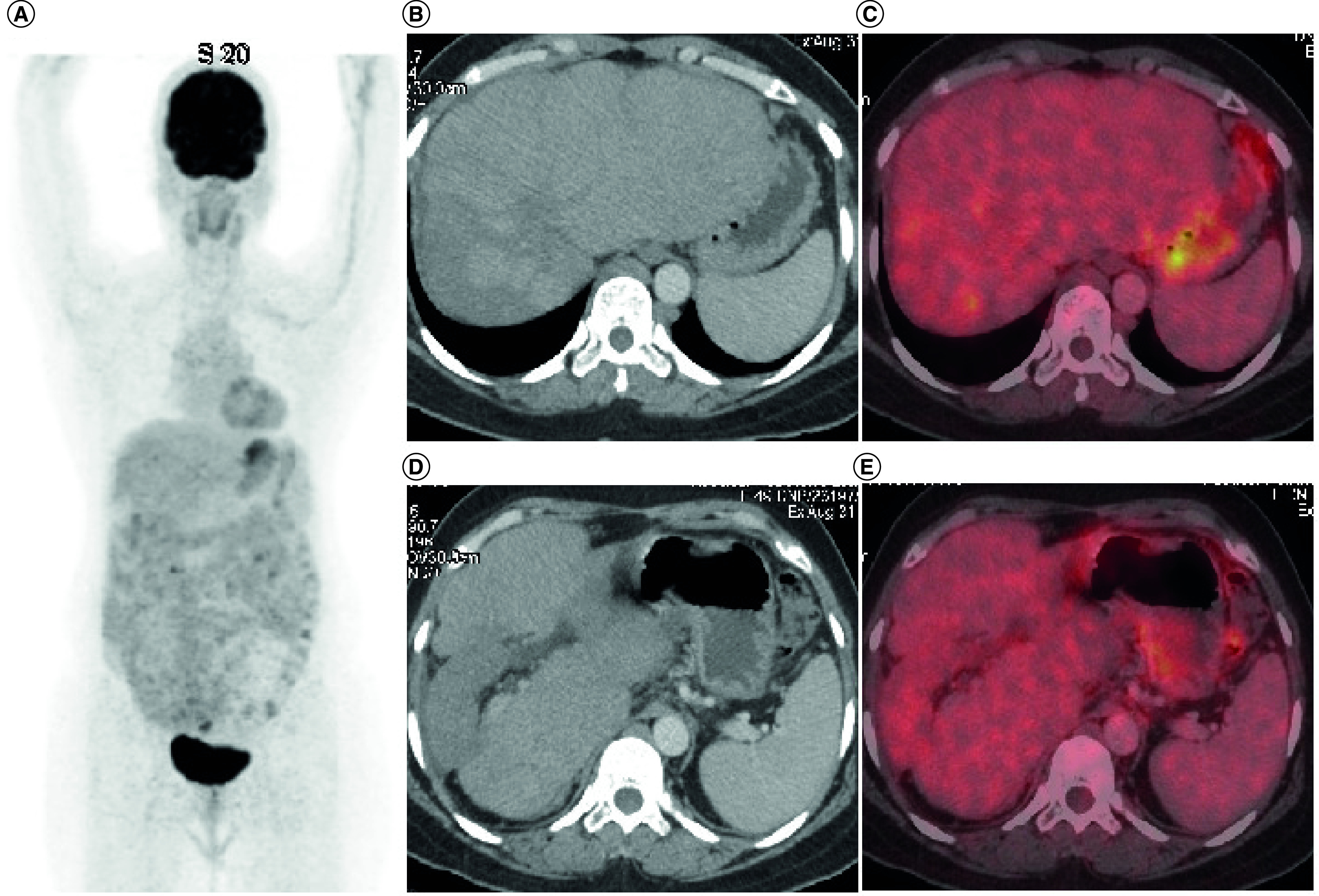
Post-treatment positron emission tomography-computed tomography scan of the 49-year-old female patient described in Figure 2 after 12 months of pazopanib-based therapy. **(A)** Maximum intensity projection image FDG PET-CT showing physiologic tracer uptake in brain, myocardium and urinary bladder and no abnormal tracer uptake in the hepatic region. **(B & D)** Axial CT abdomen showing confluent hypodense lesions in both the lobes of liver. **(C & E)** There is no significant abnormal tracer uptake in fused PET-CT images suggestive of complete metabolic response.

**Figure 4.**
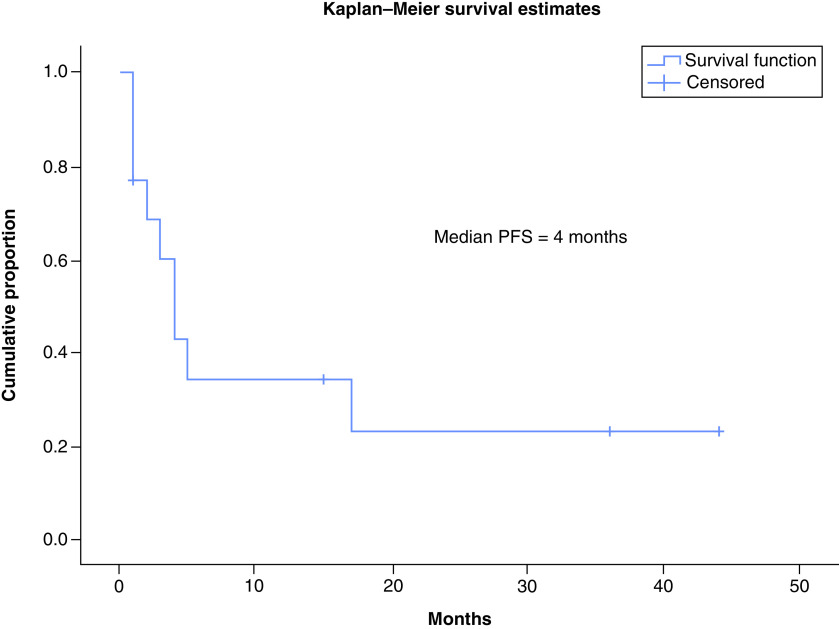
Kaplan–Meier curve showing progression free survival with first line of treatment.

**Figure 5.**
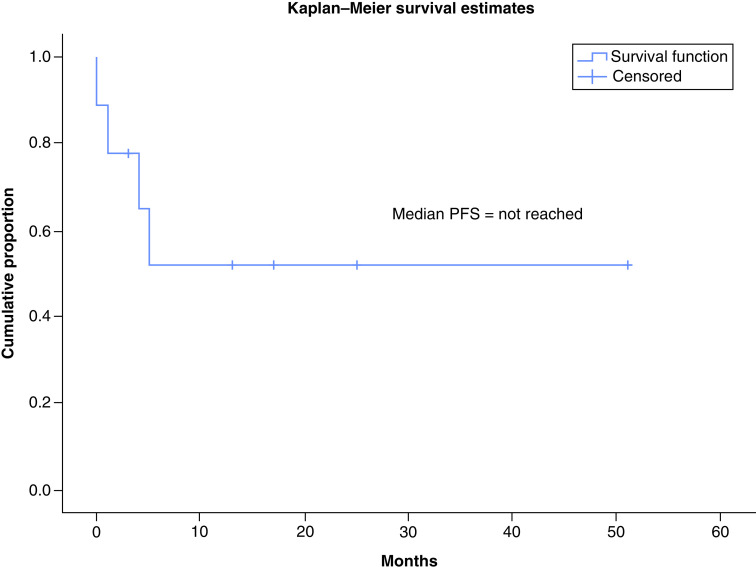
Kaplan–Meier curve showing progression free survival with tyrosine kinase inhibitors.

**Figure 6.**
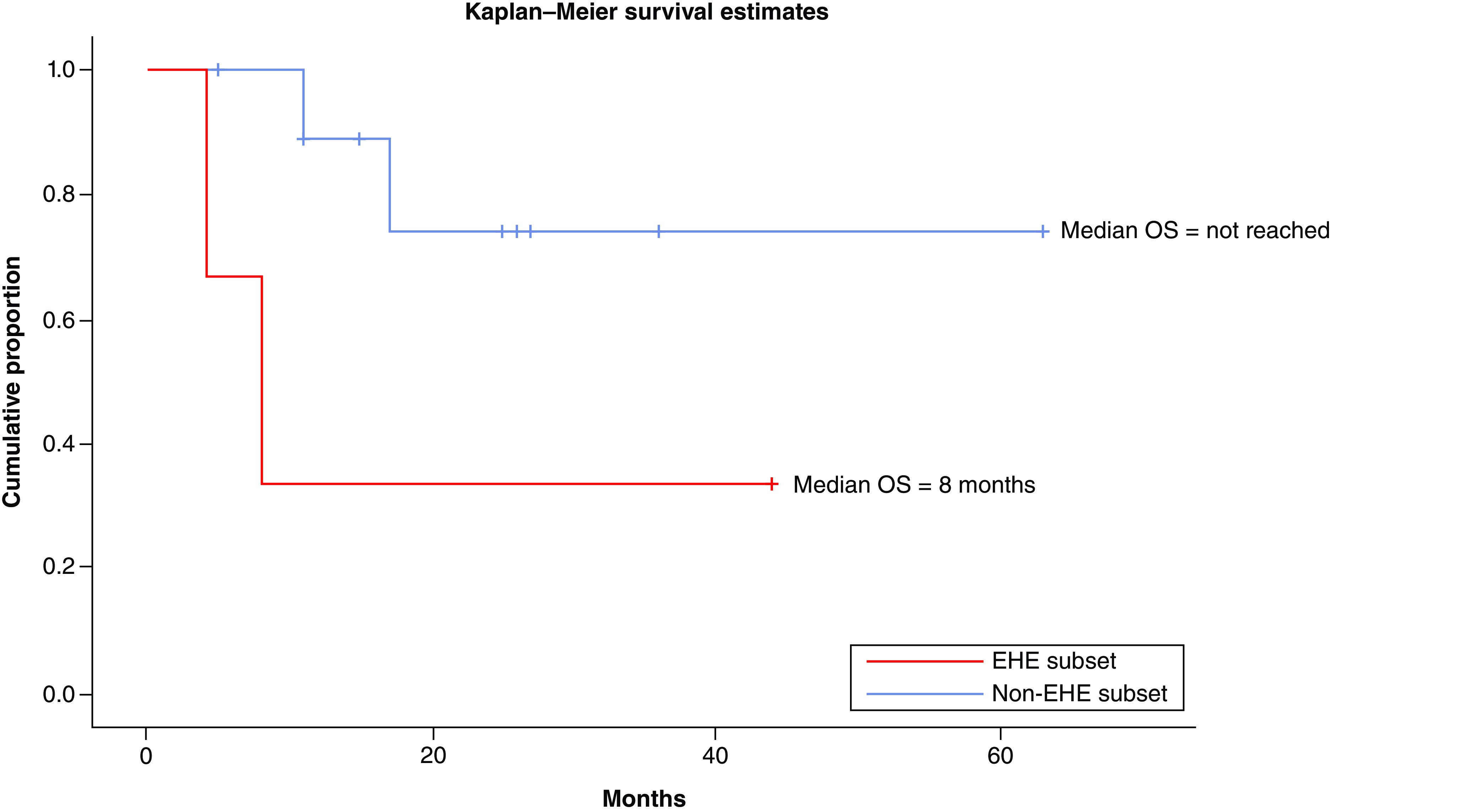
Kaplan–Meier curve showing overall survival among the epithelioid hemangioendothelioma (blue line) and non epithelioid hemangioendothelioma (red line) subsets. EHE: Epithelioid hemangioendothelioma.

TKIs produced objective response in 30% patients (three out of ten response assessments) including 20% PR (n = 2) while stable disease was attained in 20% (n = 2). A total of 10% patients (n = 1) attained CR with use of TKIs which has been radiologically depicted in Figures 2 & 3. There were no objective responses noted in patients who received conventional chemotherapy, while stable disease response has been attained in 7.7% (n = 1). The pseudomyogenic subtype was refractory to both TKI and chemotherapy, while the retiform subtype progressed to chemotherapy within 3 months followed by refractoriness to TKI. The median PFS with first-line therapy was 4 months (95% CI: 2.48–5.53) (Figure 4) while the median PFS with second line of therapy and that with TKI was not reached (Figure 5). In the overall and the EHE population, median overall survival (OS) at 20 months follow-up was not reached. In the non-EHE subgroup, the median OS was 8 months (95% CI: 1.5–14.4) but this difference between the EHE and non-EHE subgroups was not statistically significant (p = 0.06) (Figure 6). The 12-month OS was 75% (95% CI: 50-NE), while that at 20 months of median follow-up was 65% (95% CI: 37.6-NE). The 12 month PFS with first line of treatment overall was 36% (95% CI: 9–63%) and with TKIs was 58% (95% CI: 26.6–84.1%). The four deaths in the study comprised of patients of EHE (n = 2) and one patient each of pseudomyogenic and retiform subtypes.

Grade 3 or 4 adverse events noted in our patients consisted of febrile neutropenia with vinblastine, periphery sensory neuropathy with paclitaxel and hand foot skin reaction with pazopanib (n = 1; 10% each).

## Discussion

### Epithelioid hemangioendothelioma

EHE was the commonest subtype of HE in our series. The occurrence of liver as the commonest primary site is consistent with previous studies; however, the median age is younger than previously reported [8,9]. We also observed that the EHE patients in our study were predominantly male, contrary to the female preponderance reported in literature [7]. While bone is the commonest reported metastatic site in studies [10], we found lymph node to be the most frequent metastatic site in EHE. Rarer sites of metastases found in our series were pancreas and orbit. While pancreatic metastases have been scantily reported [7], orbital metastases in EHE have never been previously described in literature.

### Kaposiform hemangioendothelioma

Our patient had cutaneous and bony symptoms from infancy, which were misdiagnosed before he presented to us at the age of 3 years. He had locally advanced and infiltrative disease at the lower extremity, which corroborates with findings of previous studies [13]. A more detailed description of this case and discussion on KHE has already been published from our center [24].

### Pseudomyogenic hemangioendothelioma

Previous series describe the extremities as the most common (78%) site of disease; however, our patient had a chest wall primary which has been found in 18% patients in published literature [15]. The locally aggressive behavior of the disease with multiple local recurrences was similar to that described in past studies [25], but our patient had a more rapid tempo of disease. Following an incomplete surgical resection, the patient had disease progression within 2 months of pazopanib and doxorubicin each and eventually succumbed to his disease.

### Retiform hemangioendothelioma

Contrary to the reported description of retiform HE with primary site being extremities [17], our patient had primary disease at the spleen. Retiform splenic HE has only been rarely reported in literature, with a single case of purely retiform pathology described so far [26]. Besides this, only few reports of composite HE with epithelioid and retiform components have been published [27]. Despite classically having a low risk of metastases [17], our patient had hepatic and vertebral metastases at baseline. Spontaneous splenic rupture as first presentation of HE has been rarely reported among cases of EHE [28] and KHE [29] but no case of retiform HE presenting with splenic rupture has been described till date.

One of the largest studies on EHE was carried out retrospectively at the Royal Marsden Hospital between 1998 and 2013 with 32 patients by Agulnik *et al.* [30]. Chemotherapy (paclitaxel and anthracyclines) was used in maximum cases and did not yield any objective responses with the best response being stable disease. The use of newer agents in the last decade, such as celecoxib, imatinib, axitinib, thalidomide, pazopanib and sunitinib was also described. Median PFS was 6.7, 3.3, 1.5, 1.4 months in first, second, third and fourth lines of medical treatment, respectively. The absence of objective responses with conventional chemotherapy is similar to the findings we have obtained in our study.

Prior to presentation at our center, five patients had already undergone surgical management which included life-saving splenectomy due to splenic rupture and shock in one patient of Retiform HE. As per our protocols, we gave chemotherapy to patients with HE who presented with high volume and severe symptoms. We also added propranolol with the primary therapeutic regimen after evidence of its role in management of angiosarcoma [31]. Recent times have seen a shift from use of chemotherapy to targeted therapy in vascular sarcomas. The overall response rate to TKIs (pazopanib and sorafenib in all lines) in our study population was 30% with median PFS not reached at 20 months of follow-up. In a phase II study by Chevreau *et al.* in 15 patients with sorafenib producing median PFS of 6 months with median OS not being reached after 12 months of follow-up in a progressive EHE [32]. A 2016 retrospective review of EORTC Soft Tissue and Bone Sarcoma Group phase II and III clinical trials on pazopanib in advanced vascular sarcomas analyzed 52 patients including ten HE patients, yielding 20% response rates and median PFS of 26.3 months [33]. In a case report, Bally *et al.* described a sustained PFS of more than 100 months in metastatic hepatic EHE with pazopanib based therapy [34]. Similarly, in a patient in our study, such sustained benefit has been achieved for 63 months with pazopanib in the second line. Table 4 further lists studies that have highlighted the use of TKIs in EHE and their outcomes in comparison to our study.

**Table 4.** Comparison of previous studies of epithelioid hemangioendothelioma with this study regarding use of tyrosine kinase inhibitors for therapy.

Authors	Study type	Patients (n)	TKI used	Response	Median PFS (months)	Ref.
Cioffi A *et al.*	Retrospective	34	Sorafenib (n = 6; 17.6%)	ORR = 0% SD = 50%	4.8	[35]
			Imatinib (n = 2; 5.8% )	ORR = 0% SD = 0%		
Chevreau C *et al.*	Prospective, phase II	15	Sorafenib (n = 15)	ORR = 13% SD = 60%	6	[32]
Yousaf N *et al.*	Retrospective	19	Imatinib (n = 1; 3%)	ORR = 0% SD = 100%	NA	[36]
			Axitinib (n = 2; 6%)	ORR = 0% SD = 100%		
			Sunitinib (n = 1; 3%)	ORR = 0% SD = 0%		
			Pazopanib (n = 1; 3%)	ORR = 0% SD = 0%		
Kollar A *et al.*	Retrospective analysis of prospective trials	10	Pazopanib	ORR = 20% SD = 40%	26	[33]
Shiba S *et al.*	Retrospective	10	Pazopanib (n = 2;, 20%)	ORR = 0% SD = 100%	NA	[37]
Sparber-Sauer M *et al.*	Retrospective analysis of prospective trials	6	Pazopanib	ORR = 0%	NA	[38]
Frezza AM *et al.*	Retrospective	73	Pazopanib (n = 12; 16.4%)	ORR = 0% SD = 75%	2.9 (with pazopanib)	[39]
Current study	Retrospective	13	Sorafenib (n = 2; 15.3%)	ORR = 50% (1/2) SD = 50% (n = 1)	4	
			Pazopanib (8; 61.5%)	ORR = 25% (2/8) SD = 12.5% (n = 1)		

CR: Complete response; ORR: Overall response rate; PD: Progressive disease; PR: Partial response; PFS: Progression-free survival; SD: Stable disease; TKI: Tyrosine Kinase Inhibitors.

Stachiotti *et al.* have recently published a consensus paper on EHE [40] and have recognized it as an ultra-rare cancer which needs collaborative research efforts from the global medical community. Surgery with or without adjuvant radiotherapy has been advised for unifocal EHE with the aim for R0 resection. For metastatic disease, surgical modality should be tailored depending on the other available treatment options. Liver transplant for hepatic EHE without evidence of extrahepatic metastases has been suggested as an option. However, the EHE patient of our study, who underwent orthotopic liver transplantation outside also had distant bone metastases hence required systemic therapy eventually. Active surveillance has been advised by Stachiotti *et al.* as an acceptable option in advanced disease with low symptom burden, while systemic therapy in form of interferon, VEGF inhibitors, TKIs and chemotherapy should be given in others. The MEK inhibitor, trametinib, is a promising option under evaluation in phase II trials because MEK is overexpressed in these cases owing to CAMTA1 fusion.

The limitations of our study are its retrospective nature, small sample size and limited follow-up time. It is difficult to draw definite conclusions on the efficacy of TKIs in comparison with chemotherapy without larger, prospective studies. The phenomenon of pregnancy related EHE merits further exploration, with the background of few reports suggesting the role of hormones such as placental growth factor (PGF) in its pathogenesis [41]. The development of hepatic EHE in a treated patient of mediastinal germ cell tumor does not have a precedent in reported literature and more studies are needed to suggest its possible occurrence as a secondary malignancy. Dedicated research on other subtypes of HE is required, but is limited by the rarity of the disease.

## Conclusion

This study offers unique insights from the developing world into the clinical presentation, diagnostic challenges and treatment options for advanced HEs. Chemotherapy has only limited role and literature on newer options such as TKIs is emerging.

## Future perspective

In rare cancers such as HEs, the conduct of larger randomized control studies is a challenge. In future, there should be collaborative efforts by various international sarcoma groups to develop patient registries of these cases. Biomarkers must be recognized in the future, to predict which tumors would respond to chemotherapy or TKIs to tailor the patient’s treatment. Newer modalities of therapy should also be explored as options, such as trametinib which is under research as of now.

**Summary points**

- Hemangioendothelioma has multiple subtypes, the most common one being epithelioid hemangioendothelioma.
- Hemangioendothelioma has a spectrum of presentations, ranging from indolent behaviour to aggressive malignant disease.
- The most common primary sites are the liver and vertebrae, while the most common metastatic sites are lymph nodes and bone.
- Chemotherapy can be used for treatment of rapidly progressive and organ-threatening disease; however, documented responses to chemotherapy have been dismal.
- Tyrosine kinase inhibitors (TKIs) have a vital role in therapy and produce overall response rates of 30% (irrespective of line of therapy).
- Beta blockers can be used along with the chemotherapy or TKI agent.
- TKIs can also lead to complete remission in rare cases.
- Other anti-angiogenic agents such as bevacizumab and thalidomide can also be used for treatment.
